# Impact of contact lenses on the ocular surface microbiome, tear proteome, and dry eye disease

**DOI:** 10.1128/spectrum.02264-25

**Published:** 2026-02-04

**Authors:** Oriane S. Kopp, Sophia C. Morandi, Marco Kreuzer, Anne-Christine Uldry, Nina Eldridge, Martin S. Zinkernagel, Denise C. Zysset-Burri

**Affiliations:** 1 Department of Ophthalmology, Inselspital, Bern University Hospital, University of Bern 570140, Bern, Switzerland; 2 Interfaculty Bioinformatics Unit and Swiss Institute of Bioinformatics, University of Bern 27210, Bern, Switzerland; 3 Department for BioMedical Research, University of Bern 27210, Bern, Switzerland; Nova Southeastern University, Fort Lauderdale, Florida, USA

**Keywords:** ocular surface microbiome, contact lenses, whole-metagenome shotgun sequencing, nano liquid chromatography tandem mass spectrometry, dry eye disease

## Abstract

Although contact lens wear is widespread and known to affect the ocular surface, its impact on the ocular surface microbiome (OSM) remains poorly understood, with existing studies reporting conflicting findings. Additionally, the relationship between contact lens wear, tear proteome, and dry eye disease (DED) is unclear. In this study, we aimed to characterize the OSM (via whole-metagenome shotgun sequencing) and the tear proteome of 25 contact lens wearers and 23 age- and sex-matched controls. The dominant phyla were *Actinobacteria*, *Proteobacteria*, and *Firmicutes*, with *Cutibacterium acnes* being the most abundant species. No significant differences in microbial composition, diversity, or tear proteome were observed between contact lens wearers and controls. DED parameters (tear breakup time, Schirmer’s test, tear osmolarity, and Ocular Surface Disease Index [OSDI]) also showed no significant differences, although contact lens wearers reported a trend toward higher subjective symptoms (OSDI). Sex-stratified analysis revealed a marginal difference in microbial beta diversity between male contact lens wearers and male controls, along with increased tear production in male contact lens wearers. Female contact lens wearers reported a higher OSDI compared to female controls. These findings suggest that contact lens wear does not significantly alter the OSM or tear proteome in healthy individuals, although sex-specific responses may warrant further investigation.

**IMPORTANCE:**

Contact lenses are worn by millions of people, yet the scientific literature contains conflicting reports about their impact on the microbial communities that are naturally present on the eye surface. This study addresses these knowledge gaps by examining both the eye microbiome and tear proteins using advanced sequencing and linking them to dry eye symptoms. Understanding the relationship between contact lens wear, natural eye bacteria, and tear composition is essential for resolving contradictory findings in the field. Additionally, identifying potential sex-specific differences in how individuals respond to contact lens wear could lead to more personalized approaches to contact lens management.

## INTRODUCTION

Contact lenses, corrective lenses placed on the ocular surface, are worn by approximately 150 million people worldwide, primarily for vision correction (1). Due to their direct placement on the eye, their impact on the ocular surface has long been studied to optimize their function and comfort for users. Contact lens wear can reduce epithelial oxygen uptake (2) and disturb the tear film (3), increasing the risk of complications such as dry eye disease (DED).

However, the effect of contact lenses on the ocular surface microbiome (OSM) has been less studied. Compared to the microbiomes of the gut, the skin, and the mouth, the OSM has been less explored and was, in particular, ignored by the Human Microbiome Project (4). Recent studies suggest that there is a resident OSM with a role in the immune system and several diseases, such as DED (5, 6). However, due to mechanical (eye blinking) and chemical barriers (e.g., antimicrobial tear components), the OSM is of low microbial abundance. Thus, its characterization poses unique challenges since it is prone to contamination (7).

Existing studies on the impact of contact lens wear on the OSM report different findings. Shin et al. observed that contact lens wearers had an OSM composition more similar to skin microbiota, with significant differences compared to controls (8). In comparison, Andersson et al. found no significant differences in OSM between contact lens wearers and controls (9). Similarly, Zhang et al. found no significant differences in the OSM among controls, contact lens wearers, and orthokeratology lens wearers (10).

A limitation of these studies is the application of 16S rRNA sequencing, which only identifies bacteria. In contrast, whole-metagenome shotgun sequencing (WMSS) offers better resolution, including the detection of bacteria, eukaryotes, and fungi as well as microbial functions. To our knowledge, no study has used WMSS to investigate the OSM in contact lens wearers, nor assessed whether the OSM of contact lens wearers correlates with the tear proteome or clinical DED parameters.

Understanding how contact lens wear influences the OSM could enable identification of specific microbial species associated with contact lens-related complications such as DED and ocular irritation, informing microbiome-based interventions to prevent or treat these conditions.

To assess whether contact lens wear alters the composition and function of both the OSM and the proteome, and whether any observed alterations correlate with DED parameters, we applied WMSS and mass spectrometry to characterize the OSM and tear proteome, respectively, in contact lens wearers compared to age- and sex-matched controls. This integrated multi-omics approach, employing higher-resolution sequencing methods than previous 16S rRNA-based studies, along with clinical assessment and sex-stratified analysis, addresses methodological gaps in existing literature and enables evaluation of the relationship between contact lens wear, OSM, tear proteome, and DED parameters.

## MATERIALS AND METHODS

### Sample collection

After confirming that no exclusion criteria were met and obtaining written informed consent, conjunctival swabs were collected in the morning from 25 contact lens wearers and 23 controls using flocked nylon swabs (FLOQSwabs #518CS01, Copan, Brescia, Italy) (6, 7, 11). Sample size was calculated based on a power analysis with a power of 80% and an alpha of 0.05 (https://clincalc.com/stats/samplesize.aspx), using relative abundances of the most abundant phylum *Actinobacteria* from prior studies (6). Participants were classified as contact lens wearers if they used soft contact lenses at least 4 days per week for a minimum of 5 h per day. Before sample collection, a local anesthetic (tetracaine 1%, Théa, Schaffhausen, Switzerland) was applied for participant comfort, as topical anesthetics do not affect overall sequencing results (12). Swabs from both eyes were pooled for each participant. To ensure quality, five negative controls, flocked nylon swabs treated with a drop of tetracaine 1%, were processed alongside the samples.

Additionally, tear fluid was collected using a Schirmer’s test type 1. A standard filter strip (Haag-Streit AG, Köniz, Bern, Switzerland) was positioned in the lower conjunctival bag of both eyes. After 5 min, the stripes were removed and immediately processed for tear fluid extraction as described in (13).

### Metagenomic DNA sequencing

Conjunctival swabs and negative controls were processed on the same day for DNA extraction using the QIAamp DNA Microbiome Kit (51704, QIAGEN, Hilden, Germany). Extracted DNA was stored at −20°C until further analysis. Sequencing libraries were prepared using the NEBNext Ultra II Preparation Kit (SKU E7645L) and sequenced on an Illumina NovaSeq 6000 platform for 150 paired-end cycles at the Next-Generation Sequencing Platform of the University of Bern, Switzerland.

Sequencing reads contained Unique Molecular Identifiers (UMIs [14]), which were extracted using UMI-tools (v. 1.1.4). Quality filtering was performed with fastp (v. 0.20.1 [15]), followed by mapping to the human reference genome GRCh38 to remove host DNA. Unmapped reads were extracted using SAMtools (v. 1.10) and subsequently mapped to the ChocoPhlAn reference database (mpa_vOct22_CHOCOPhlAnSGB_202212) using Bowtie2 (v. 2.3.4.1 [16]). The resulting SAM files were converted to BAM format, sorted, and indexed using SAMtools (v. 1.10 [17]). Reads were deduplicated based on UMIs and mapping locations using UMI-tools (v. 1.1.4). The deduplicated BAM files were analyzed using the MetaPhlAn4 (v. 4.0.4 [18, 19]) pipeline, while deduplicated FASTQ files were processed with HUMAnN3 (v. 3.8 [20]) for functional profiling.

Potential contaminants were identified using the decontam R package (v. 1.26.0 [21]), applying the prevalence-based method and a classification threshold of 0.3. To refine the contaminant list, a PubMed search was conducted via the rentrez package (v.1.2.3 [22]). Taxa were retained if mentioned in at least five articles referencing either ocular microbiome or conjunctiva microbiota, or if their species name and one of the keywords “eye”, “conjunctiva”, or “ocular” were present in the article title or abstract. The objective of this literature-based filtering step was to preserve taxa plausibly present on the OSM but that could have been misclassified as contaminants, like skin bacteria (Fig. S1).

### Tear fluid processing and analysis

The extracted tear fluid was stored at −80°C until further analysis via nano liquid chromatography tandem mass spectrometry (nLC-MS/MS). Sample preparation, SDS-PAGE separation, in-gel digestion, and nLC-MS/MS were performed as previously described in reference 11. Tear fluid proteins were denatured in urea/Tris buffer, reduced with DTT, alkylated with IAA, and quenched prior to SDS-PAGE. Gel lanes were sectioned and subjected to in-gel enzymatic digestion. The digests were analyzed by liquid chromatography on a Dionex Ultimate 3000 (Thermo Fisher) coupled to a QExactive HF mass spectrometer (Thermo Fisher Scientific) with one injection of 5 μL digests. Peptides were trapped on a µPrecolumn C18 PepMap100 (5 μm, 100 Å, 300 μm × 5 mm, Thermo Fisher Scientific, Reinach, Switzerland) and separated by backflush on a C18 column (3 μm, 100 Å, 75 μm × 15 cm, Nikkyo Technos, Tokyo, Japan) by applying a 40 min gradient of 5% acetonitrile to 40% in water, 0.1% formic acid, at a flow rate of 350 nL/min. The Full Scan method was set with a resolution at 60,000 with an automatic gain control (AGC) target of 1E06 and a maximum ion injection time of 50 ms. A top 15 data-dependent method for precursor ion fragmentation was applied with the following settings: resolution 15,000, AGC of 1E05, maximum ion time of 110 ms, mass window 1.6 *m*/*z*, collision energy 27, under fill ratio 1%, charge exclusion of unassigned and 1+ ions, and peptide match preferred, respectively. MS data were processed as described in reference 11 using FragPipe (v20.0) and MSFragger (v3.8), with searches conducted against the Human SwissProt database. Label-free quantification (MaxLFQ) was carried out using IonQuant, and data processing included peptide- and protein-level filtering at a 1% false discovery rate (FDR), variance stabilization normalization, and renormalization of MaxLFQ after contaminant removal, and Gaussian-based imputation of missing values.

### DED parameters

Four clinical assessments of DED, the following parameters were measured: the Ocular Surface Disease Index (OSDI), TearLab Osmolarity Test, tear breakup time (TBUT), and Schirmer’s test I.

The OSDI is a validated questionnaire with 12 questions that assess dry eye symptoms, visual function, and environmental triggers over the past week. It is a copyrighted instrument of AbbVie Inc. (©2020 AbbVie. All rights reserved. Used with permission). Tear osmolarity was measured using the TearLab Osmolarity Test, following the manufacturer’s protocol (https://trukera.com/tearlab/, accessed on 25 June 2023). The TBUT was measured using BioGlo Fluorescein Sodium Ophthalmic Strips U.S.P. It was recorded using a slit lamp as the time from the last blink to the appearance of the first dry spot on the cornea. Schirmer’s test I was performed as described above.

The samples were taken in the following order: OSDI, TearLab Osmolarity, Schirmer’s test I, conjunctival swab, and TBUT.

### Statistical analysis

#### Demographic data

All statistical analyses were performed using R software (v. 4.4.2 [23]). Demographics were compared among groups applying Fisher’s exact test (for sex), Welch’s *t*-test (for tear osmolarity), or Mann-Whitney *U* test (for age, TBUT, Schirmer’s test, and OSDI score; Table 1).

**TABLE 1:** Characteristics of study participants^*a*^

Feature	CL	CN	*P* value CL vs CN
Males (*n*)	9	11	0.6
Age (year)	26.16 ± 4.21	29.43 ± 8.88	0.06
TBUT (s)	11.71 ± 6.05	11.13 ± 4.92	0.97
Schirmer’s test I (mm)	29.11 ± 8.55	27.23 ± 8.51	0.32
Tear osmolarity (mOsmol/L)	290.74 ± 6.64	290.29 ± 7.66	0.83
OSDI score	13.98 ± 5.68	11.92 ± 3.48	0.11

^*a*^ Data are presented as mean ± standard deviation. Group comparisons were performed using Fisher’s exact test (for categorical data [sex]), Mann-Whitney *U* test (non-normally distributed variables [age, TBUT, Schirmer’s test I, and OSCDI score]), and Welch’s *t*-test (normally distributed data [tear osmolarity]). CL, contact lens wearers (*n* = 25), CN, controls (*n* = 23).

#### Sequencing data

**Fig 1:**
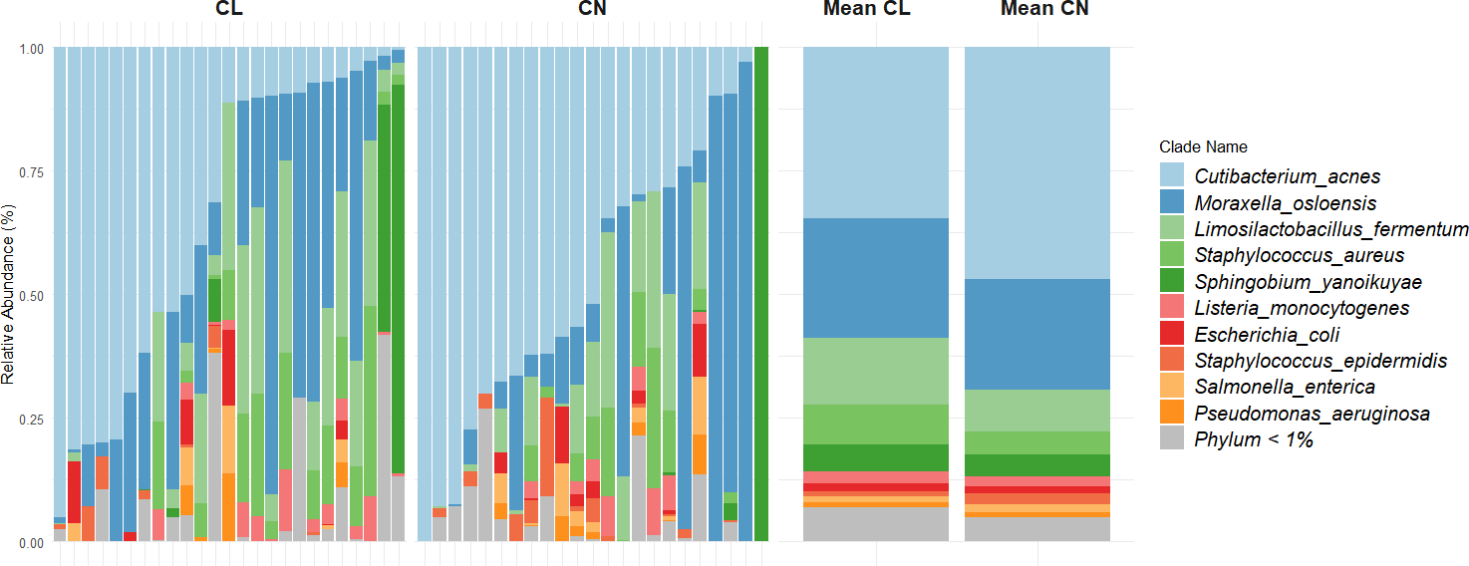
Taxonomic characterization of the ocular surface microbiome at the species level. Relative abundances of microbial taxa for individual participants, stratified by groups (left), and mean values for groups (right; CL, contact lens wearers [*n* = 25]; CN, controls [*n* = 23]).

**Fig 2:**
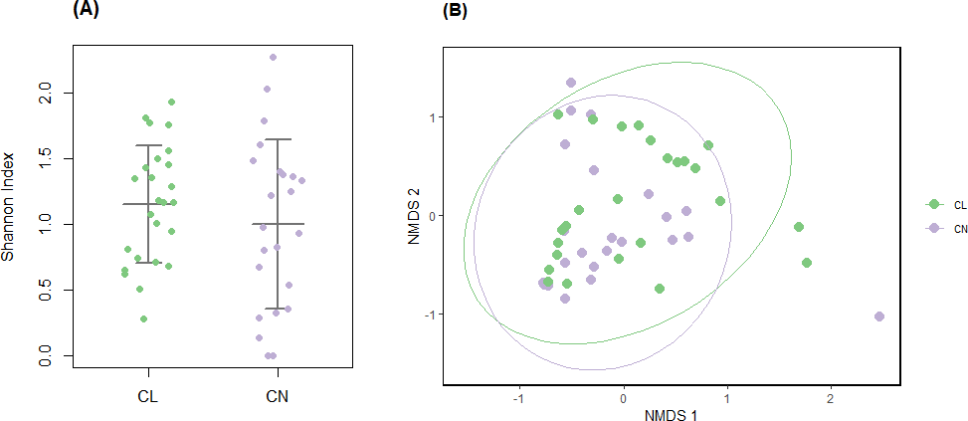
Diversity of the ocular surface microbiome. Alpha diversity, measured using the Shannon (**A**) index, was compared between contact lens wearers and controls. No significant differences were observed (*P* = 0.35, Welch’s *t*-test). Each dot represents an individual participant. Horizontal lines indicate group means ± standard deviation. Beta diversity using non-metric multidimensional scaling (NMDS) with Bray-Curtis dissimilarity index (**B**) was used to visualize differences in microbial composition at the species level between the groups. Ellipses represent 95% confidence intervals. No significant separation between the groups was observed (*P* = 0.22, *F* = 1.45, *R*^2^ = 0.031, permutational multivariate analysis of variance). CL, contact lens wearers (*n* = 25) and CN = controls (*n* = 23).

Microbial composition was visualized for individual samples, grouped by contact lens wearers and controls, using the R package ggplot2 (v. 3.5.1 [24]; Fig. 1), along with group means. Alpha diversity was assessed using the Shannon index, calculated with the R package vegan (v. 2.6.10 [25]), and group comparison was performed using Welch’s *t*-test (Fig. 2A).

To evaluate global differences in microbial and pathway abundances, non-metric multidimensional scaling (NMDS) with Bray-Curtis dissimilarity index was conducted using the vegan and ggplot2 packages (Fig. 2B). Group separation significance was tested via permutational multivariate analysis of variance (PERMANOVA) with 999 permutations using the vegan R package.

Association between microbial taxa/pathway abundances and contact lens usage was assessed using multivariate association by linear models from the package MaAsLin2 (v. 1.20.0 [26]). Significant associations were defined as those with a *q*-value < 0.25 and presence in at least 50% of the samples, after FDR adjustment (Benjamini–Hochberg). “DNA isolation kit number” and “sex” were included as random effects and “contact lens wear status” as fixed effect. Input data were log2-transformed with a pseudo count added (minimum non-zero value divided by 2) to account for zeros.

Differential abundance analysis was performed using ZicoSeq (implemented in the GuniFrac R package, v. 1.8 [27]). The model compared microbial abundances between groups with “DNA isolation kit number” and “sex” as covariates. Statistical significance was assessed using a permutation-based FDR threshold of 0.1 (999 permutations; Fig. S2A).

Scoring system for DED parameters, given that no participants met established criteria for DED, a composite score was developed to evaluate variations in DED parameters. For each parameter, the observed range within the study population was divided into five equal intervals, with each interval assigned a score from 1 to 5, reflecting the severity of DED. For TBUT, the range was from 4.0 to 23.5 s, for OSDI 0–25 points, Schirmer’s test I 8–35 mm, and tear osmolarity 279.0–308.5 mOsm/L.

The direction of scoring depends on the indicator; for some parameters, higher values suggest greater dry eye severity (OSDI, tear osmolarity), while for others, lower values indicate worse conditions (TBUT, Schirmer’s test; Fig. 5). Welch’s *t*-test was used to compare the score between the groups.

#### Tear proteome

Differential expression analysis was conducted using the empirical Bayes method with *P*-value adjustments performed using the Benjamini-Hochberg method. The statistical significance threshold for differential expression was set at an adjusted *P*-value of 0.05 and a minimum log2 fold change of 1 in absolute value (see Fig. 7A). No proteins met the significance criteria, and thus, none were retained for further individual analysis.

A principal component analysis (PCA) was conducted on MaxLFQ protein intensities, and group differences were assessed using PERMANOVA (see Fig. 7B). The DAVID bioinformatics tool (28, 29) was used to perform functional annotation of all identified proteins (see Fig. 8).

#### Sex-stratified analysis

**Fig 3:**
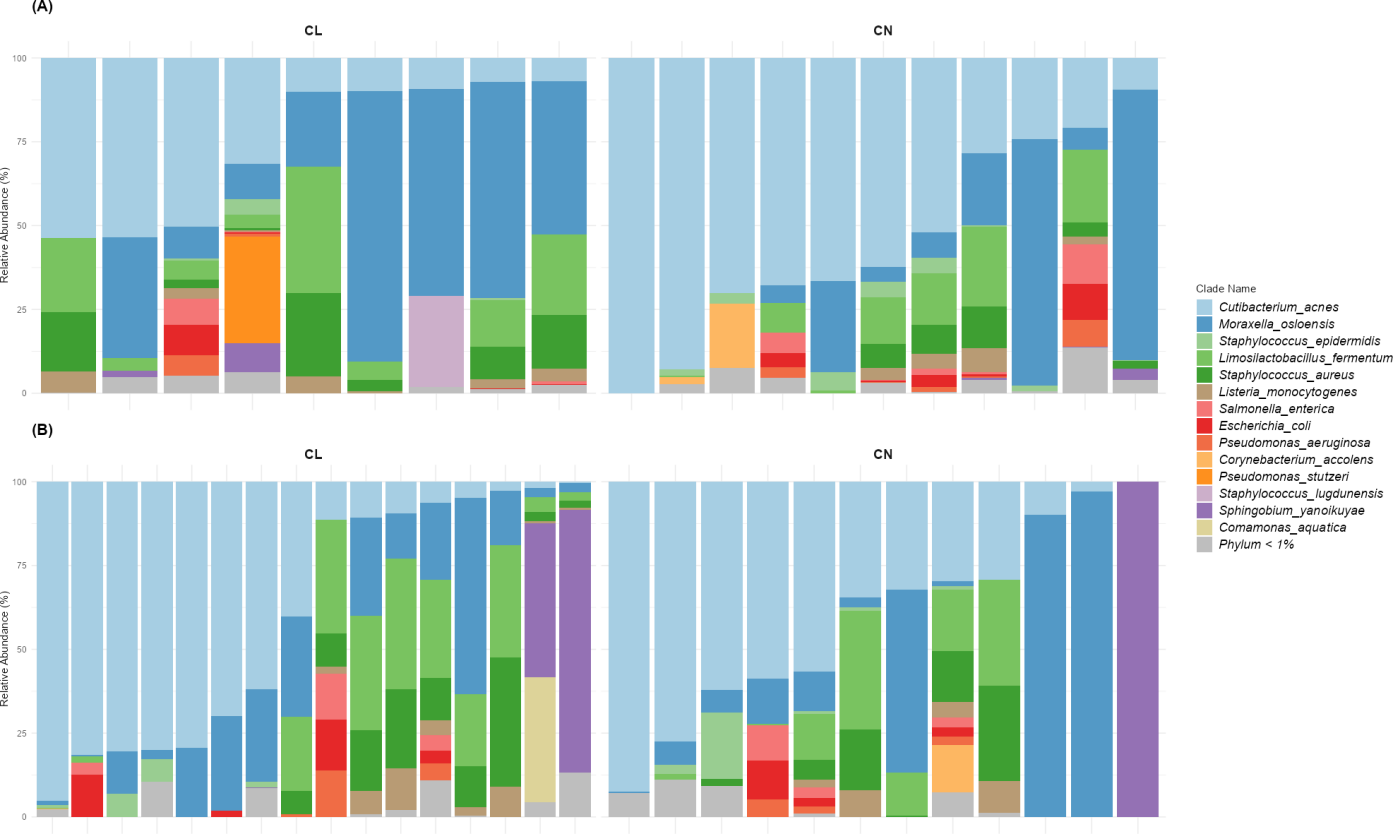
Sex-stratified taxonomic characterization of the ocular surface microbiome. Relative abundances of microbial species for (**A**) males (CL = contact lens wearers, *n* = 9; CN = controls, *n* = 11) and (**B**) females (CL, *n* = 16; CN, *n* = 12).

**Fig 4:**
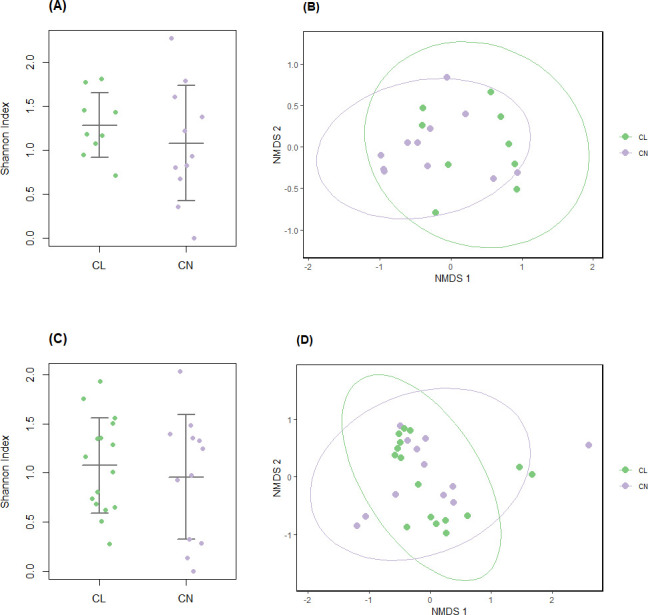
Sex-stratified diversity of the ocular surface microbiome. Alpha diversity based on the Shannon index was compared between contact lens wearers and controls in (**A**) males and (**C**) females. No significant differences were observed (*P* = 0.39 for males and 0.6 for females, Welch’s *t*-test). Each dot represents an individual participant. Horizontal lines indicate group means ± standard deviation. Beta diversity using NMDS with Bray-Curtis dissimilarity index to visualize differences in microbial composition at the species level for (**B**) males and (**D**) females between contact lens wearers and controls. Ellipses represent 95% confidence intervals. There was a marginally significant difference in the male subgroup (*P* = 0.056, *F* = 2.68, *R*^2^ = 0.13), suggesting a possible effect at the 10% significance level. No significant difference was observed in the female subgroup (*P* = 0.89, *F* = 0.27, *R*^2^ = 0.01, PERMANOVA). CL, contact lens wearers (males, *n* = 9; females, *n* = 16); CN, controls (males, *n* = 11; females, *n* = 12)

Sex-stratified analyses were performed to compare contact lens wearers and controls within male and female subgroups. These included composition plots (Fig. 3), PERMANOVA, alpha and beta diversity (Fig. 4), MaAsLin2, ZicoSeq (Fig. S2B and C), DED parameters analysis (see Fig. 6), and tear proteome analysis (Fig. S4).

## RESULTS

### Demographic data

A total of 48 conjunctival swabs were sequenced from 25 participants who were wearing contact lenses daily and 23 participants who never or only occasionally wore contact lenses in the previous months. The two groups were sex and age matched (Table 1). The contact lens wearers were wearing contact lenses for between 3 months and 18 years, with a median of 10 years.

### Taxonomic characterization of the OSM in contact lens wearers

In total, 6.85 billion 150 bp paired-end reads with an average insert size of 350 bp were generated, with an average of 142.76 ± 25.47 million reads per sample. As expected and described in previous studies (7), 87.42% of these reads were of human origin. After trimming and host filtering, we kept 836.54 million high-quality, non-human reads with an average of 17.43 ± 9.18 million reads per samples. After removing duplicate reads using UMIs, we had a total of 0.82 million deduplicated reads with an average of 0.017 ± 0.015 (mean ± standard deviation) million reads per sample for further analysis. Due to the high number of reads detected in negative controls after filtering, on average 0.28 ± 0.35 million reads per control, decontamination was performed as described in Materials and Methods, removing 29 species identified by MetaPhlAn4.

The microbiome composition was mostly bacterial (99.73% ± 1.54%), with the remainder being fungi. The most abundant phyla were *Actinobacteria* (41.93% ± 32.50 %), *Proteobacteria* (35.16% ± 30.27%), and *Firmicutes* (22.64% ± 24.17%). At the class level, *Actinomycetia* (41.93% ± 32.50%) and *Gammaproteobacteria* (28.13% ± 26.56%) were the most present. The most abundant genera were *Cutibacterium* (40.61% ± 31.34%), *Moraxella* (23.18% ± 27.34%), and *Limosilactobacillus* (11.19% ± 13.06%; mean values ± standard deviation). *Cutibacterium acnes* was the dominant species, present in 47 of the 48 samples. A total of 58 species were identified (Fig. 1). Among them, only four were present in at least 50% of the samples.

Alpha diversity was assessed using the Shannon index and compared between contact lens wearers and controls (Fig. 2A). No significant differences were observed between the groups (*P* = 0.35, Welch’s *t*-test). Beta diversity through NMDS revealed no separation between the two groups (*P* = 0.22, *F* = 1.45, *R*^2^ = 0.031, PERMANOVA; Fig. 2B).

Multivariate analysis revealed no significant associations between microbial abundance and contact lens use at the species level nor at any higher taxonomic level. Similarly, ZicoSeq did not reveal any taxa with significantly different abundances between the groups (Fig. S2A).

Common contact lens-associated contaminants, *Pseudomonas* and *Staphylococcus aureus* (30), were analyzed, but no significant differences in their relative abundances were found between contact lens wearers and controls based on both, Mann-Whitney *U* test (*P* ≥ 0.14) and MaAsLin2 analysis (*q* ≥ 0.58; Fig. S3).

### Taxonomic characterization of the OSM in contact lens wearers in males and females

Since DED is more abundant in females (31), sex-specific differences in the OSM between contact lens wearers and controls were explored.

For both sexes, the microbiome composition was mostly bacterial (99.96% ± 0.12% for males and 99.95% ± 0.27% for females). The most abundant phyla were *Actinobacteria* (43.34% ± 31.59% for males and 40.93% ± 33.67% for females), *Proteobacteria* (35.41% ± 28.33% for males and 35.35% ± 31.80% for females), and *Firmicutes* (21.21% ± 19.45% for males and 23.67% ± 27.35% for females). At the class level, *Actinomycetia* (43.34% ± 31.59% for males and 40.93% ± 33.67% for females) and *Gammaproteobacteria* (33.46% ± 27.34% for males and 24.32% ± 25.80% for females) were the most present. The most abundant genera were *Cutibacterium* (41.35% ± 29.50% for males and 40.08% ± 33.22% for females), *Moraxella* (27.87% ± 29.24% for males and 19.82% ± 26.02% for females), and *Limosilactobacillus* (10.05% ± 10.95% for males and 12.01% ± 14.53% for females; mean values ± standard deviation). *C. acnes* was the dominant species, present in all male samples and in 27 of the 28 female samples. A total of 39 species were identified in males and 48 in females (Fig. 3).

Alpha diversity was assessed using the Shannon index and compared between contact lens wearers and controls for both sexes (Fig. 4A and C). No significant differences were observed between the groups (*P* = 0.39 for males and 0.60 for females, Welch’s *t*-test). Beta diversity through NMDS revealed no separation between contact lens wearers and control in females; however, there was a marginally significant difference in the male subgroup (*P* = 0.056, *F* = 2.68, *R*^2^ = 0.13; Fig. 4B), suggesting a possible effect at the 10% significance level. No significant difference was observed in the female subgroup (*P* = 0.89, *F* = 0.27, *R*^2^ = 0.01, PERMANOVA; Fig. 4D).

Multivariate analysis revealed no significant associations between microbial abundance and contact lens use for both sexes. Similarly, ZicoSeq did not reveal any taxa with significantly different abundances between the groups (Fig. S2B and C) in males and females.

Common contact lens-associated contaminants, *Pseudomonas* and *S. aureus* (30), were also analyzed, but no significant differences in their relative abundances were found between contact lens wearers and controls in male and female subgroups based on both, Mann-Whitney *U* test (*P* ≥ 0.088 for males and ≥0.61 for females) and MaAsLin2 analysis (*q* ≥ 0.79 for males and ≥0.87 for females; Fig. S3).

### DED parameters in contact lens wearers

**Fig 5:**
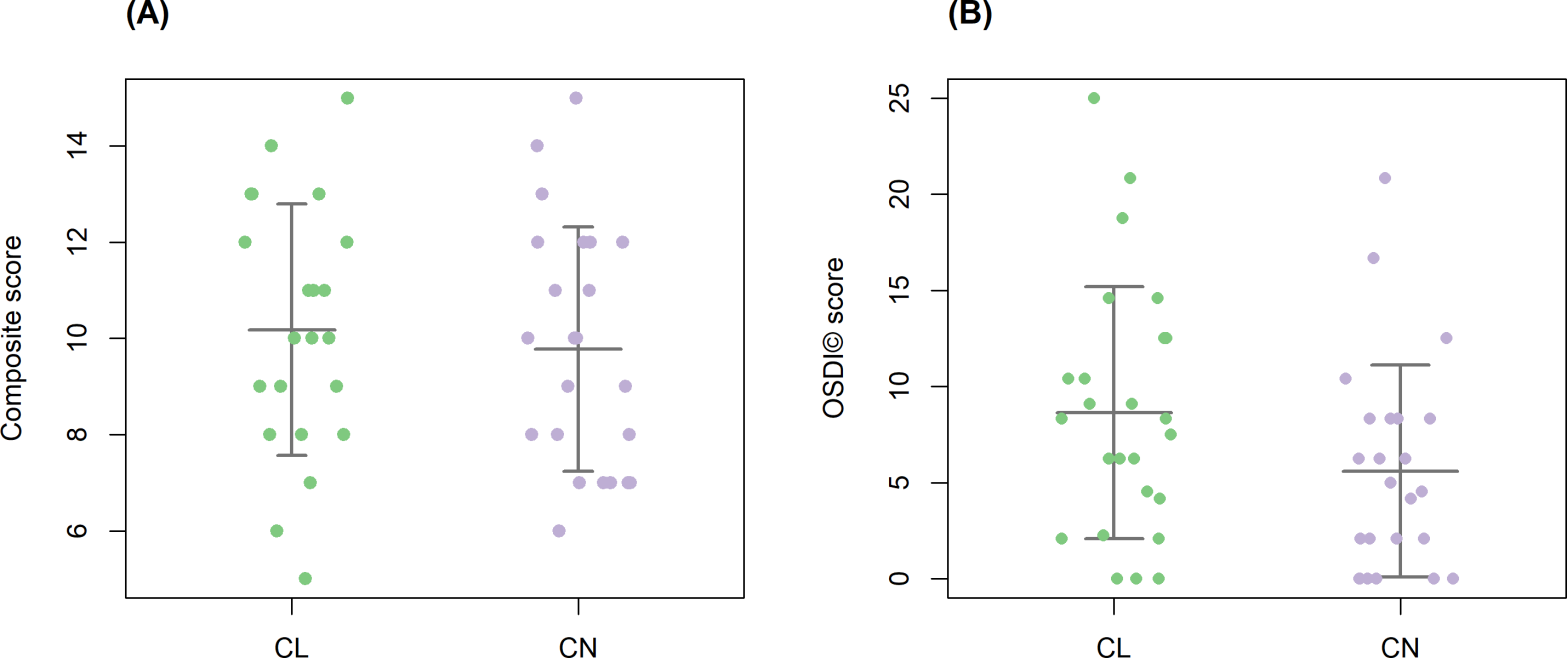
Dry eye diseae (DED) scores. (**A**) A composite DED score, integrating TBUT, Schirmer’s test I, tear osmolarity, and OSDI score, was compared between contact lens wearers (CL; *n* = 22; three study participants were excluded due to the absence of at least one parameter) and controls (CN;, *n* = 23). Welch’s *t*-test revealed no significant difference between groups (*P* = 0.61). (**B**) OSDI score was compared between groups. The OSDI score approached but did not reach the significance level (*P* = 0.068, Mann-Whitney *U* test). Each dot represents an individual participant. Horizontal lines indicate group means ± standard deviation.

Clinical parameters of DED were compared between contact lens wearers and controls. These were integrated into a composite DED score as described in Materials and Methods (Fig. 5A). Statistical analysis showed no significant difference between the two groups (*P* = 0.61, Welch’s *t* test).

When assessed individually, none of the DED parameters showed significant differences between groups, but OSDI scores (Fig. 5B) approached statistical significance (*P* = 0.068, Mann-Whitney *U* test).

### DED parameters in contact lens wearers in males and females

**Fig 6:**
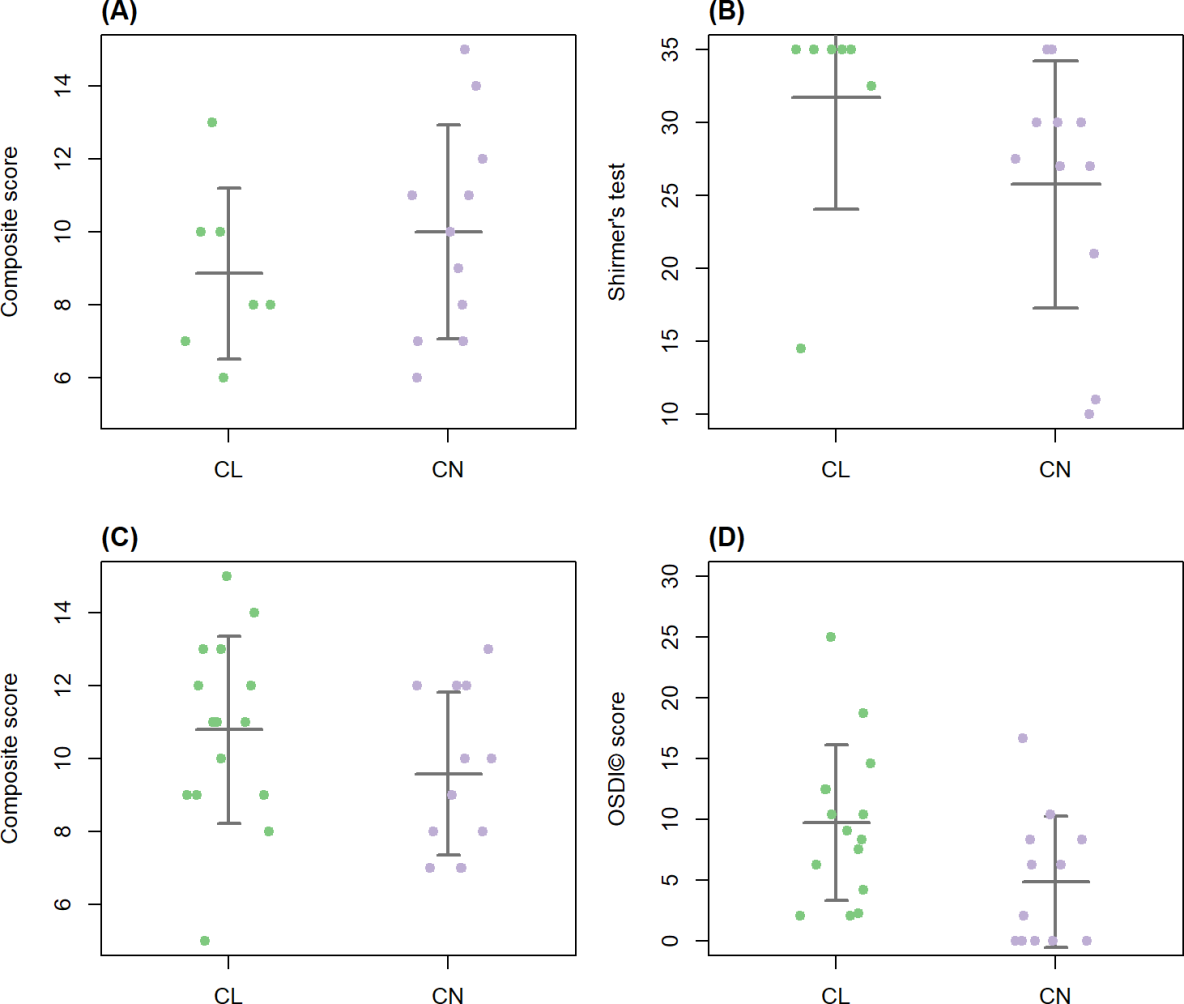
Sex-stratified dry eye disease (DED) scores. A composite DED score, integrating TBUT, Schirmer’s test l, tear osmolarity, and OSDI score, was compared between contact lens wearers and controls in (**A**) males and (**C**) females. No significant differences were observed (*P* = 0.38 for males and 0.2 for females, Welch’s *t*-test). The same analysis performed on all DED parameters revealed a significant difference in the Schirmer’s test in the male subgroup (*P* = 0.04, Welch’s t test) (**B**) and in the female subgroup a significant difference in the OSDI (*P* = 0.029, Mann-Whitney *U* test) (**D**). Each dot represents an individual participant. Horizontal lines indicate group means ± standard deviation. CL, contact lens wearers (males, *n* = 7; females, *n* = 15); CN, controls (males, *n* = 11; females, *n* = 12).

Clinical parameters of DED were compared between contact lens wearers and controls in males (Fig. 6A) and females (Fig. 6C) using a composite DED score. Statistical analysis showed no significant difference between the two groups (*P* = 0.38 for males and 0.2 for females, Welch’s *t* test).

When we assessed the DED parameters individually, in the male subgroup, Schirmer’s test I was significantly higher in contact lens wearers (*P* = 0.04, Welch’s *t* test; Fig. 6B), and in the female subgroup, the OSDI was significantly higher in contact lens wearers (*P* = 0.029, Mann-Whitney *U* test; Fig. 6D).

### Functional characterization of the OSM in contact lens wearers

Functional pathway profiles of microbes were identified for 17 samples. The limited number of annotated pathways reflects the low microbial biomass characteristic of the OSM, as HUMAnN3 requires sufficient microbial sequence depth for pathway reconstruction. The majority of the pathways were either unmapped (50.02% ± 1.73%) or unintegrated (49.41% ± 1.72%). Only four pathways were detected in at least 15% of the samples (Table 2).

**TABLE 2:** Functional profiling of the ocular surface microbiome^*a*^

Pathway	Mean abundance	*N* (non-zero)
PWY-6823: molybdopterin biosynthesis	0.2568%	14
PWY-5686: UMP biosynthesis I	0.1317%	6
SER-GLYSYN-PWY: superpathway of L-serine and glycine biosynthesis I	0.0289%	5
LIPASYN-PWY: phospholipases	0.0079%	3

^*a*^ Microbial pathways detected in at least 15% of the samples.

A PERMANOVA analysis revealed no significant difference in pathway composition between contact lens wearers and controls (*P* = 0.66). Similarly, MaAsLin2 did not identify any pathways associated with contact lens use.

Due to the reduced number of samples, this analysis was not performed on the male and female subgroups.

### Functional characterization of the tear proteome in contact lens wearers

**Fig 7:**
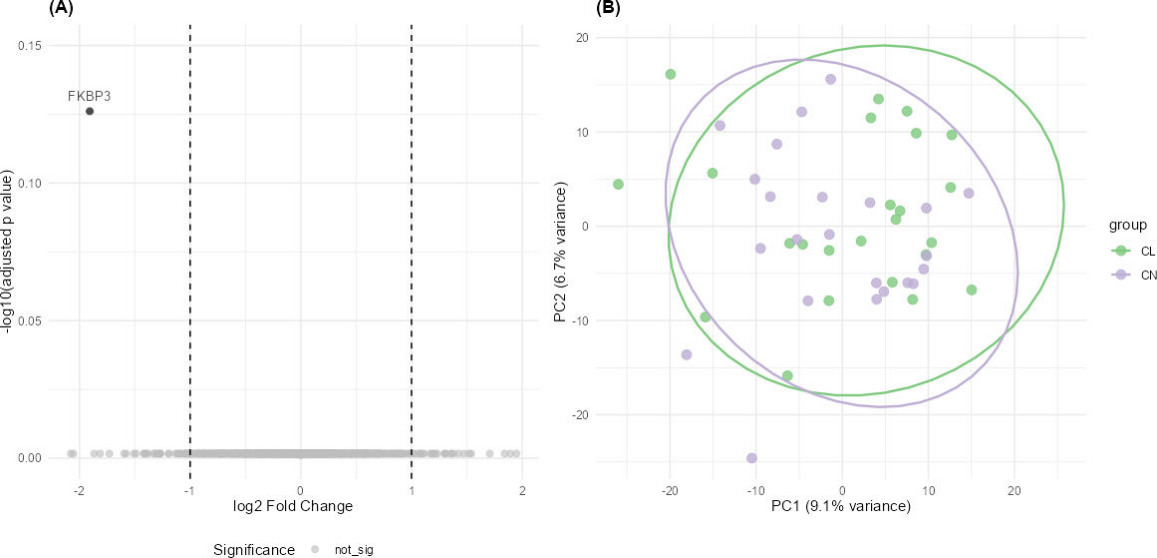
Characterization of the tear proteome. (**A**) Volcano plot of the 1,078 identified proteins, comparing expression in contact lens wearers and controls. No proteins met significant thresholds (adjusted *P* < 0.05 and |log2 fold change| ≥ 1), with FKBP3, peptidyl-prolyl *cis-trans* isomerase, having the smallest adjusted *P*-values (*P* = 0.74). (**B**) PCA of the tear proteins showed no separation between groups (*P* = 0.76, *F* = 0.9, *R*^2^ = 0.02, PERMANOVA; CL = contact lens wearers, *n* = 24; CN = controls, *n* = 23).

A total of 1,078 proteins were identified in tears of contact lens wearers and controls. None of them were significantly differentially regulated between the groups (adjusted *P* value < 0.05 and fold change ≥ 2 or ≤−2; Fig. 7). The protein with the lowest adjusted *P*-value was peptidyl-prolyl *cis-trans* isomerase FKBP3 with an adjusted *P*-value of 0.74 and a log2FC of −1.9. No separation between groups based on protein expression was found (*P* = 0.76).

The same analysis performed on the male and female subgroups revealed no protein significantly differentially expressed in contact lens wearers and controls (Fig. S4A and C) nor a separation between groups (*P* ≥ 0.65, PERMANOVA; Fig. S4B and D).

**Fig 8:**
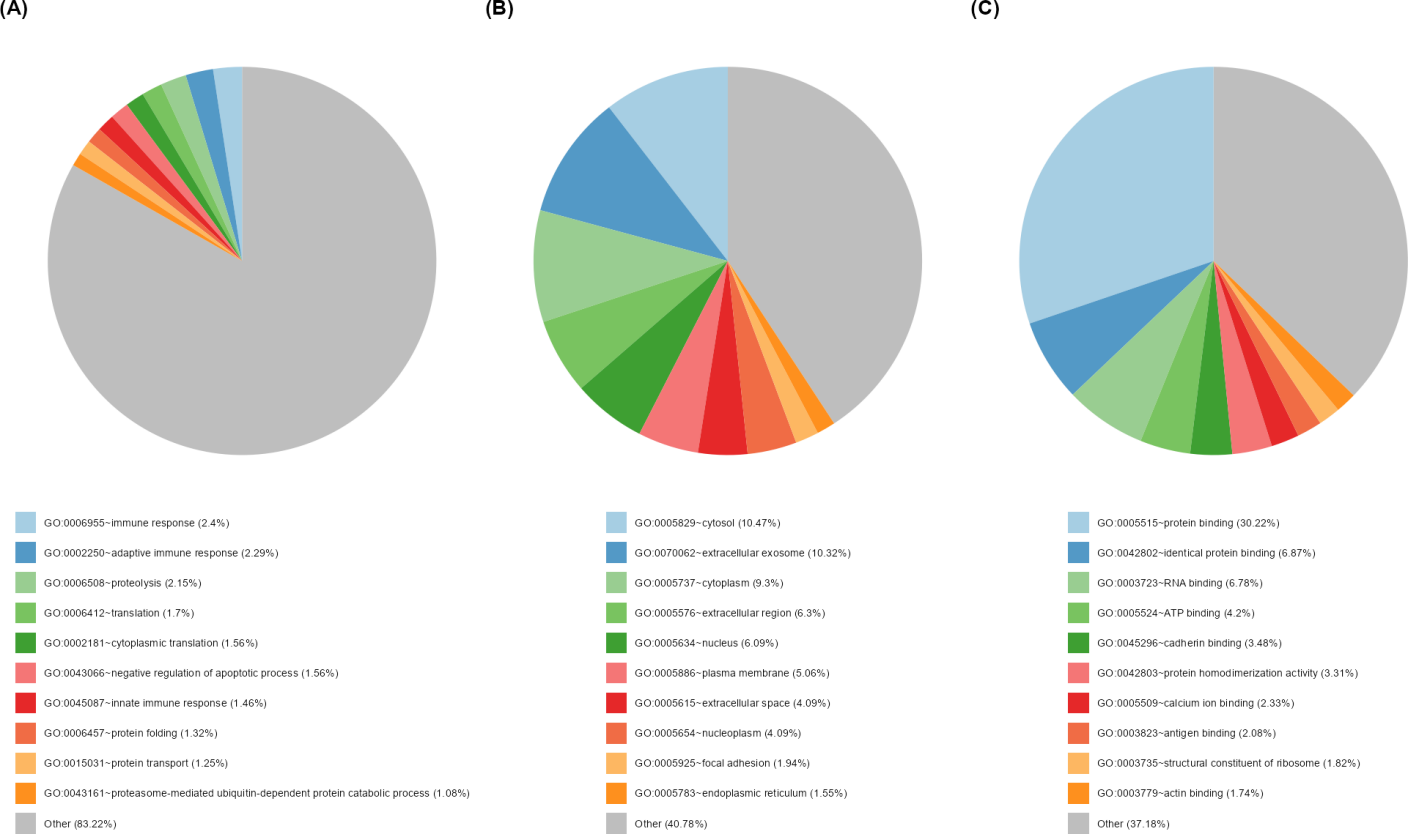
Functional annotation of the tear proteome. Functional classification of the tear proteins (*n* = 1,078) based on gene ontology (GO) categories (**A**) biological processes, (**B**) cellular components, and (**C**) molecular functions using the DAVID bioinformatics tool.

Using the DAVID bioinformatics tool, functional annotation revealed that the tear proteome was mainly involved in the molecular function of protein binding (30.5%), in the biological process in adaptive immune response (2.8%). These activities take place mainly in the cellular components, cytosol (10.6%), extracellular exosome (10.3%), and cytoplasm (9.5%; Fig. 8).

## DISCUSSION

Corrective lenses placed on the ocular surface for visual correction alter the ocular surface (2) and the tear film (3). However, the impact of contact lenses on the OSM is less studied, and existing studies have reported conflicting findings (8–10). Microbial imbalance in the OSM could lead to dysbiosis associated with several diseases, including DED (6). In this study, we aimed to explore the OSM, the tear proteome, and clinical parameters of DED in contact lens wearers compared to age- and sex-matched controls.

### The OSM in contact lens wearers

The overall microbial composition of the OSM was similar between contact lens wearers and controls, consistent with previous studies showing a predominance of *Actinobacteria*, *Proteobacteria*, and *Firmicutes* at the phylum level (5, 13).

All analyses performed on the OSM (alpha and beta diversity measurements, MaAsLin2 and ZicoSeq; Fig. 1 and 2; Fig. S2) resulted in no significant difference between contact lens wearers and controls. These findings are consistent with those reported by Andersson et al. (9) and Zhang et al. (10) but differ from the results of Shin et al. (8). This discrepancy is likely due to differences in study design. In the study by Shin et al., participants were sampled at three time points (approximately every 2 weeks), and all samples were included in the comparative analysis, potentially introducing dependence among observations from the same individual. In contrast, our study involved a single sampling per participant, resulting in fewer total samples but ensuring statistical independence. Additionally, the inclusion criteria for defining contact lens wearers in Shin et al. were not clearly reported, which may further contribute to differences between studies.

The sex-stratified analysis revealed no difference in alpha diversity for both sexes, but a marginal difference in beta diversity among male participants, suggesting a potential sex-specific effect (Fig. 4). However, this difference was not explained by any species identified through MaAsLin2, and no similar trend was observed in females. Given the small subgroup sample size, further studies with larger cohorts are needed to explore whether sex modulates the impact of contact lens wear on the OSM.

Functional profiling of the OSM revealed few annotated microbial pathways, reflecting the low microbial biomass on the ocular surface. Among the four identified pathways, serine/glycine biosynthesis (SER-GLYSYN-PWY) and phospholipase biosynthesis (LIPASYN-PWY) may be relevant for ocular diseases. Serine plays a crucial role in retinal metabolism, and its availability is essential to maintain retinal structure and function (32). Phospholipases, such as phospholipase D, are involved in the ocular response to stress (33). These findings suggest that microbial functions such as serine/glycine biosynthesis and phospholipase production may influence ocular surface homeostasis. Dysregulation of these pathways could contribute to the development of DED by promoting epithelial stress, inflammation, and tear film instability (34).

### DED in contact lens wearers

We found no significant differences in TBUT, Schirmer’s test, tear osmolarity, nor the composite DED score between contact lens wearers and controls (Fig. 5A). These findings are unexpected, since contact lens use has often been associated with ocular surface irritation and increased symptoms of dryness in previous reports (35). A potential explanation is that the contact lens wearers in our cohort represent individuals who have adapted well to lens use and are therefore less likely to exhibit clinical signs of DED.

However, the OSDI scores showed a non-significant trend of higher values in contact lens wearers compared to controls (Fig. 5B), suggesting a difference between subjective symptoms (feeling of dry eyes) and objective clinical signs. This observation may be influenced by various factors, including contact lens types, materials, and duration of use. This discrepancy between clinical assessment and subjective feeling is known in DED research (36). It also raises the possibility that contact lens wearers may experience dry eye sensations that are not detectable through clinical tests. Further studies, with larger cohorts, are needed to determine whether contact lens use increases dry eye symptoms, even in the absence of clinical signs of dry eyes.

The sex-stratified analysis revealed no statistically significant differences in the DED composite score between contact lens wearers and controls in either male or female subgroups (Fig. 6A and C). However, male contact lens wearers have significantly higher Schirmer’s test I values compared to male controls (Fig. 6B), suggesting increased tear production in this subgroup. In contrast, female contact lens wearers showed significantly higher OSDI scores relative to female controls (Fig. 6D), indicating a greater subjective symptom associated with contact lens use in females.

The increased Schirmer’s test I values observed in male contact lens wearers may reflect a compensatory tear production response to dryness induced by the contact lenses. However, this hypothesis requires validation in larger studies specifically designed to assess sex-related differences in DED and contact lens use. The finding of higher OSDI scores in female contact lens wearers, compared to female controls, suggests that contact lens use may increase DED symptoms in females. It is plausible that the augmented tear production observed in males may mitigate the perception of dryness associated with contact lens use, potentially accounting for the absence of increased OSDI scores in this subgroup.

### The tear proteome in contact lens wearers

The absence of differentially expressed proteins between contact lens wearers and controls suggests that, in healthy individuals, contact lens wear does not significantly alter tear protein composition, regardless of sex. This aligns with the absence of microbial and clinical differences observed in the cohort. However, these findings do not correlate with previous studies, which found differences in tear proteome between contact lens wearers and controls (37). As previously stated, the patients in this cohort might have adapted to their contact lenses and thus expressed no differences in tear proteome composition either.

Peptidyl-prolyl *cis-trans* isomerase FKBP3, the protein with the lowest adjusted *P*-value in our data set, is known to be involved in immunoregulatory and intracellular signaling processes (38) and is expressed in retinal tissue (39). However, it did not meet statistical significance, and its potential relevance to tear physiology remained unclear. Further studies are needed to determine whether FKBP3 plays a role in adaptation to contact lens wear and the underlying biological mechanisms.

Functional annotation of the tear proteome revealed enrichment in GO term categories that were consistent with previous studies (6).

### Relationship between OSM, tear proteome, and DED in contact lens wearers

The absence of significant differences in OSM composition, tear proteome, and DED parameters between contact lens wearers and controls suggests that well-adapted contact lens wearers maintain ocular surface homeostasis. This finding has important implications for understanding the mechanisms underlying contact lens-associated discomfort. The absence of microbial dysbiosis or tear proteome alterations, despite a trend toward higher subjective symptoms (OSDI), suggests that contact lens discomfort in adapted contact lens wearers arises primarily through mechanical rather than microbiological or biochemical mechanisms. This contrasts with other forms of DED, such as meibomian gland dysfunction, where OSM dysbiosis and tear proteome alterations have been documented (6). In contact lens wearers, mechanical factors such as friction between the lens and the cornea may be the dominant causes of symptoms, while the OSM and tear proteome adapt to maintain stability.

### Limitations

The small sample size, particularly for sex-stratified analyses, limits statistical power. The sex-stratified results may only suggest trends and necessitate validation in larger-scale studies. Additionally, the low microbial biomass of the OSM poses challenges in detecting taxonomic or functional differences. An important limitation is the inclusion of contact lens wearers with highly variable wear duration (range: 0–18 years, median: 10 years), which may explain the lack of significant differences in OSM composition, tear proteomics, and DED parameters compared to controls. This heterogeneity might mask some effects, as long-term wearers may have adapted to contact lens wear, while newer wearers may still be undergoing changes. The variation in wear duration prevents the detection of time-dependent effects that may be present at specific stages of adaptation. Future studies should employ longitudinal designs with repeated measurements at multiple time points in cohorts of new wearers to capture dynamic changes during the adaptation period. Although contact lens wear was carefully defined, including daily wear status, future studies should explore longer-term effects, lens material properties, and hygiene practices in more detail. Sequencing of lenses themselves could provide complementary information.

The contamination removal approach, while based on published OSM literature, introduces some circularity as earlier studies may not have employed equally rigorous decontamination methods. However, this conservative approach was necessary to preserve known ocular-associated bacteria that might otherwise be removed as contaminants. Importantly, both study groups underwent identical processing and contamination removal, ensuring that any systematic effects would affect both groups equally. Future studies would benefit from larger, well-characterized negative control data sets and the development of standardized contamination databases specific to low-biomass ocular samples.

A key strength of this study is the use of WMSS, which enables high-resolution profiling beyond 16S rRNA sequencing, and the precise definition of contact lens wear.

### Conclusion

In this study, contact lens wear did not significantly alter the OSM, tear proteome, or clinical parameters of DED in healthy individuals. However, sex-specific trends, such as increased tear production in males and higher subjective dry eye symptoms in females, suggest differential responses to lens wear that warrant further investigation. These findings highlight the importance of considering sex as a biological variable in ocular surface research involving contact lenses.

## Data Availability

The data sets supporting the conclusions of this article are available in the European Nucleotide Archive under the accession numbers PRJEB67995 and PRJEB97939.

## SUPPLEMENTAL MATERIAL

Supplemental figures spectrum.02264-25-s0001.pdf Figures S1 to S4.

ASM does not own the copyrights to Supplemental Material that may be linked to, or accessed through, an article. The authors have granted ASM a non-exclusive, world-wide license to publish the Supplemental Material files. Please contact the corresponding author directly for reuse.
