# Supplementary material for: Impact of contact lenses on the ocular surface microbiome, tear proteome, and dry eye disease
Source: Microbiol Spectr. 2026 Feb 4;14(3):e02264-25. doi: 10.1128/spectrum.02264-25 (PMC12955385; doi:10.1128/spectrum.02264-25)
Supplement: Supplemental figures — Figures S1 to S4. [file spectrum.02264-25-s0001.pdf]

Supplemental material

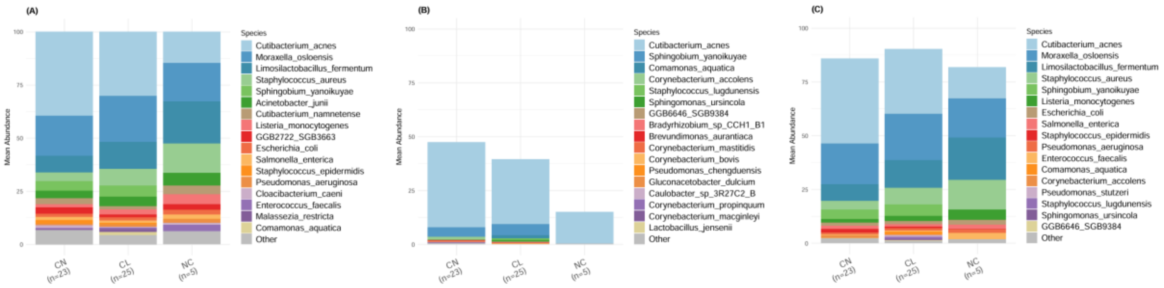

Figure S1: Taxonomic characterisation of the ocular surface microbiome at species level at different filtering steps.

Taxonomic characterisation before filtering (A), after applying the decontam tool using the prevalence method with a threshold of 0.3 (B), and after retrieving species classified as contaminants from decontam using an automatized literature search in PubMed (C). CL = contact lens wearers (n = 25), CN = controls (n = 23), NC = negative controls (n = 5).

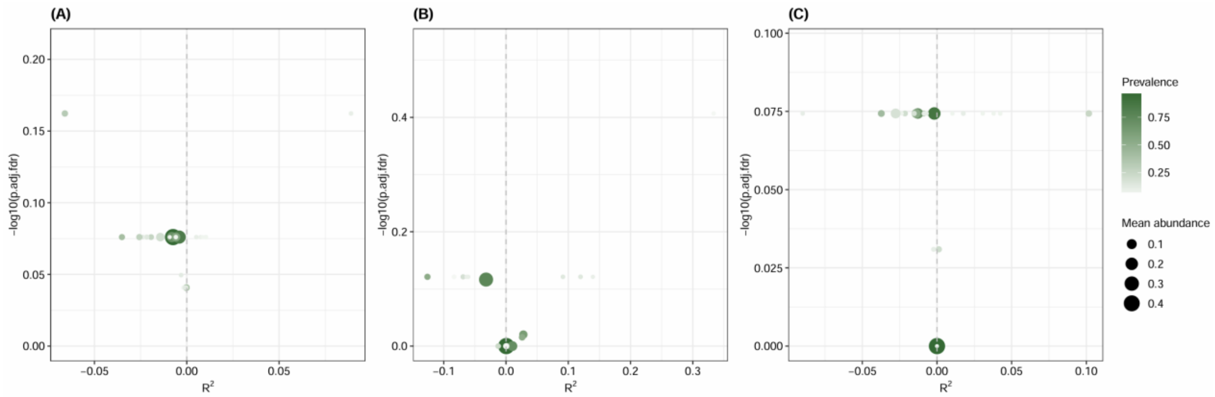

Figure S2: Differential abundance analysis

Differential abundance analysis performed using ZicoSeq between contact lens wearers (n = 25; males, n= 9; females, n = 16) and controls (n = 23; males, n= 11; females, n = 12) in the whole dataset (A), male (B) and female subgroups (C). No species passed the significant threshold (adjusted p-values < 0.1).

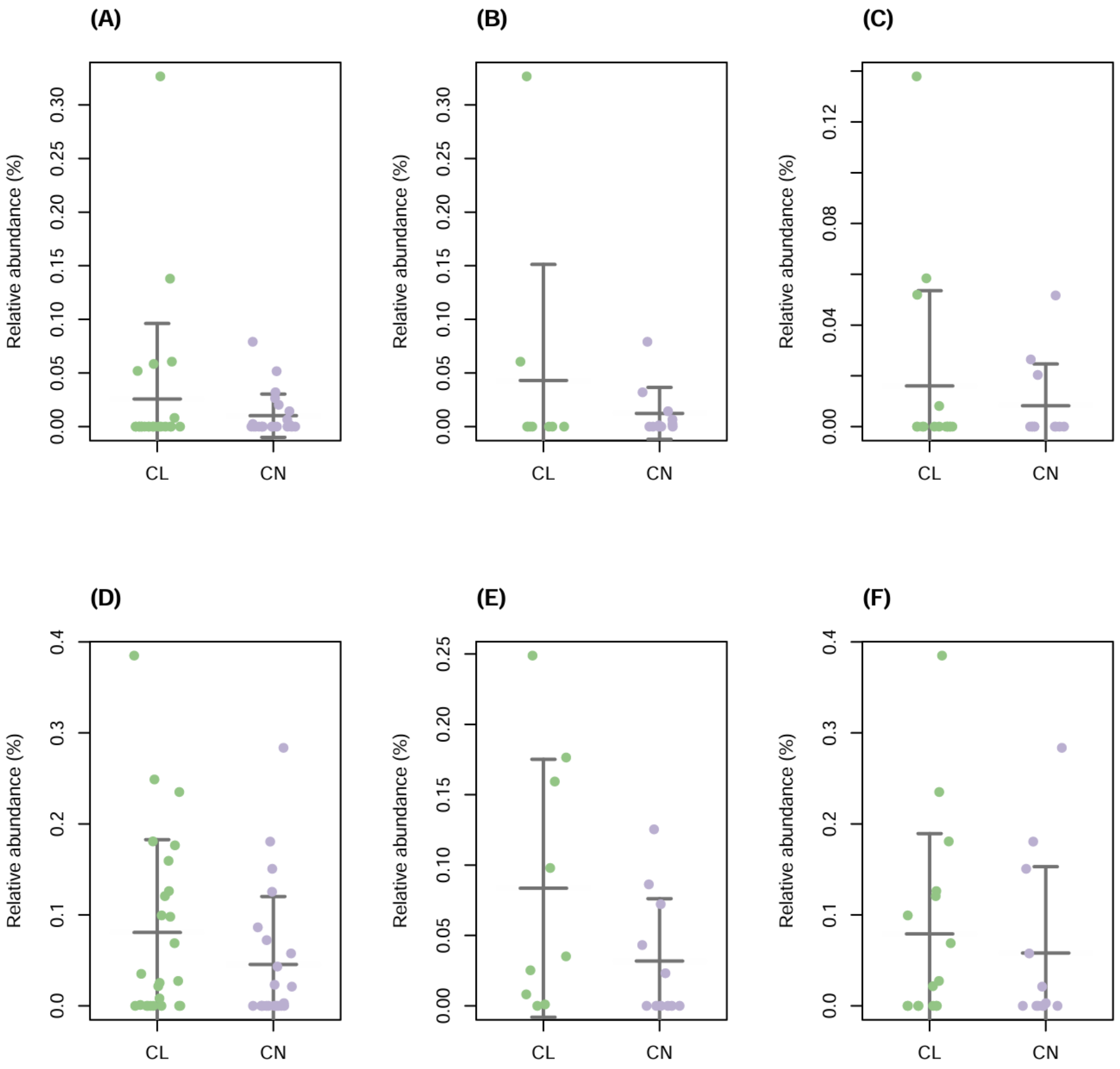

Figure S3: Abundances of known contact lens contaminants in groups.

Pseudomonas and *Staphylococcus aureus* are known contaminants of contact lenses and related accessories (Thakur and Gaikwad, 2014). Pseudomonas in the whole dataset (A), males (B) and females (C) are presented, as well as *Staphylococcus aureus* in all (D), males (E) and females (F). Differences between groups were tested using the Mann-Whitney U test but none were significant (p values ≥ 0.14 in the whole dataset, ≥ 0.088 in males and ≥ 0.61 in females). To correct for multiple testing, FDR-adjusted q-values were also calculated using MaAsLin2, resulting in values of ≥ 0.58 for the whole dataset, ≥ 0.79 in males and ≥ 0.87 in females. These results indicate that neither taxon showed a statistically significant difference in abundance between contact lens wearers and controls nor in male and female subgroups. CL = contact lens wearers (n = 25; males, n= 9; females, n = 16), CN = controls (n = 23; males, n= 11; females, n = 12).

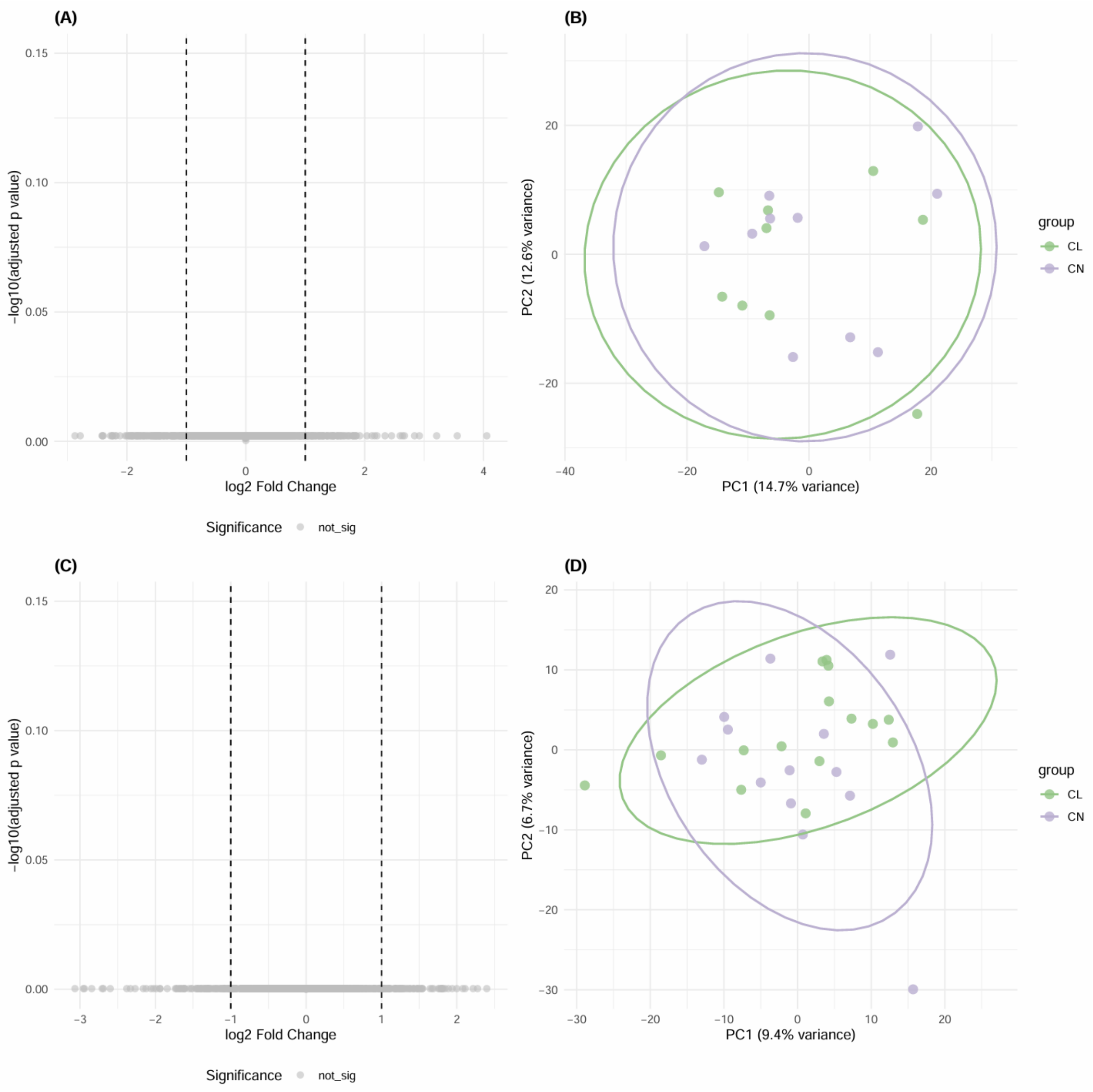

Figure S4: Sex stratified functional characterisation of the tear proteome.

Volcano plots of the 1078 identified proteins, comparing expression in contact lens wearers and controls for males (A) and females (C). No proteins met significant thresholds (adjusted $p < 0.05$ and $|\log2 \text{ fold change}| \geq 1$). Principal Component Analysis (PCA) of the tear proteins showed no separation between groups in males (B, $p = 0.89$) and females ($p = 0.65$, PERMANOVA; CL = contact lens wearers, n = 24; CN = controls, n = 23).
